# Evidence for repeated failure of the giant Yigong landslide on the edge of the Tibetan Plateau

**DOI:** 10.1038/s41598-020-71335-w

**Published:** 2020-09-01

**Authors:** Changbao Guo, David R. Montgomery, Yongshuang Zhang, Ning Zhong, Chun Fan, Ruian Wu, Zhihua Yang, Yingying Ding, Jijun Jin, Yiqiu Yan

**Affiliations:** 1 Institute of Geomechanics, Chinese Academy of Geological Sciences, Beijing, 100081 China; 2 Key Laboratory of Active Tectonics and Crustal Stability Assessment, China Geological Survey, Beijing, 100081 China; 3 Department of Earth and Space Sciences, University of Washington, Seattle, WA 98195 USA; 4 China University of Geosciences (Beijing), Beijing, 100083 China

**Keywords:** Environmental sciences, Natural hazards

## Abstract

Field surveys and radiocarbon dating of detrital materials provide evidence that repeated landslides dammed the Yigong Tsangpo River ca. 3500 bc, 1300 bc, 1000 bc, 600 bc, and twice more recently. Together with historical slides in 1900 and 2000, these six older slides make for a total of eight known channel-damming landslide events at the same location over the past six millennia, indicating sub-millennia recurrence intervals over this time period. Together with the likely incomplete nature of the sedimentary record of past channel-damming episodes uncovered to date, our findings indicate late Holocene multi-century-scale recurrence intervals for large landslides at this location. Hence, the riverbed at and immediately upstream of this location may have been inundated by sediment, and therefore not incising, for much of the post-glacial period. Together with the location of this landslide complex at the head of the major knickzone defining the fluvial edge of the Tibetan Plateau, our findings support the hypothesis that repeated glacial and landslide damming in this region inhibited headward propagation of river incision into the Tibetan Plateau.

## Introduction

Landslides can be important erosional processes in upland landscapes with moderate to steep hillslopes^1^. In particular, landsliding dominates hillslope erosion in rapidly uplifting terrain where high-relief, threshold hillslopes, and relatively narrow river gorges are common^1–5^. Landslides can introduce large amounts of sediment into river systems and large landslides can dam channels, impounding water and sediment^6,7^. In turn, an elevated sediment supply can retard river incision by shielding bedrock^8^. Korup and Montgomery proposed that frequent glacial damming in the eastern Himalayan syntaxis (EHS) during the Quaternary inhibited headward incision of major rivers, thereby retarding dissection into the edge of the Tibetan Plateau^2^.

While this hypothesis was rooted in evidence for repeated glacial damming of the Yarlung Tsangpo immediately above its gorge through the Himalaya^9^, similar glacial and landslide blockages occur on other major tributaries upstream of the deeply incised bedrock gorges in the EHS^2^. Little is known, however, about the frequency with which channel damming events have occurred in the region. The Lulang landslide-dammed lake in the upper reaches of the Lulang River, a tributary to the Parlung Tsangpo, was reported recently to have remained stable from before 24.2 ka bp to around 8.8 ka bp^10^. This period extends roughly through the last glacial maximum and well into the early Holocene. Like the glacial dam on the Yarlung Tsangpo, the Lulang landslide sits just upstream of the headward extent of the knickzone upstream of the deeply incised-trans-Himalayan bedrock gorge^10^.

In April, 2000, the giant Yigong landslide dammed the Yigong Tsangpo River with 3 × 10^8^ m^3^ of sediment that formed a 60 m high dam^11^. Subsequent failure of the dam sent floodwaters rushing down through the gorge, causing extensive damage for 500 km downstream into India^12^. Like the glacial dam at the head of the Tsangpo Gorge, and the landslide dam on the Lulang River, the Yigong landslide is also located immediately upstream of the knickzone defining the headward limit of river incision into the edge of the Tibetan Plateau. Here we present evidence for older landslides at the site of the Yigong Landslide, documenting repeated damming of the river at this location over the late Holocene. Our findings show that repeated failures of large landslides at this location both present a recurrent hazard and help retard headward propagation of fluvial knickpoints, thereby affecting the development of river profiles in the EHS^13^.

## Field area

**Figure 1:**
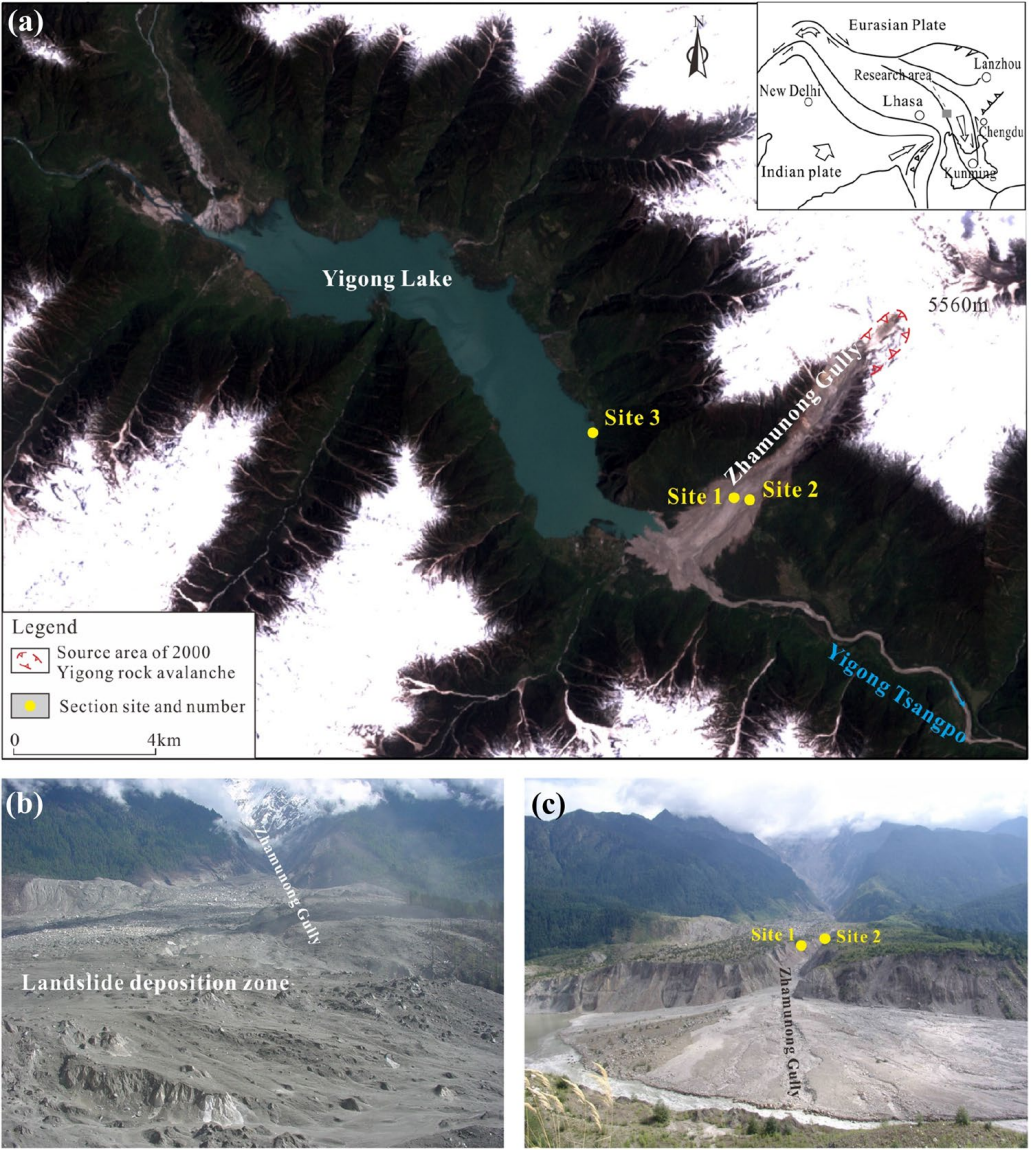
Location map of the Yigong landslide section at 30° 10′ 58″ N, 94° 55′ 53″ E. (**a**) Landsat-7 image obtained on May 4, 2000 (25 days after Yigong landslide occurred (image data download from https://www.gscloud.cn, which is open and free sharing for science study); (**b**) photograph of Yigong distal debris avalanche deposition zone before the dam failed (provided by Y.P. Yin, taken on April 23, 2000); (**c**) photograph of Yigong landslide after the 2000 dam broke (photo by Guo in 2014, view to NE). Figure generated with CorelDRAW X7 software (from https://www.coreldraw.com).

The giant Yigong landslide occurred in Zhamunong Gully, a tributary of Yigong Tsangpo River 59 km upstream of the confluence with the Yalu Tsangpo River (Fig. 1a). The area is underlain by Gangdisi bedrock^14^, with Himalayan granites exposed in the upper part of Zhamunong gully. The active Jiali-Chayu fault passes through the landslide area, which together with active faulting along the nearby Lulang-Yigong fault left the rock mass in the landslide area highly fractured.

On April 9, 2000, a rock avalanche^1^ of about 3 × 10^7^ m^3^ collapsed from a hilltop at 5,500 m in elevation and dropped more than 1,500 m, impacting older detrital materials deposited along Zhamunong gully^11^. The resulting debris avalanche transformed into a high-speed debris flow that swept both sides of the Zamunong valley as it ran out 8 km in under 10 min before depositing^15^ (Fig. 1b). The Yigong Tsangpo was completely blocked as the landslide formed a natural dam with a length of 4.6 km, a width of up to 3 km, a thickness of 55–110 m, resulting in a deposit with a total volume of about 3 × 10^8^ m^3^^11,16,17^. The dammed lake collapsed 2 months later^18^, producing a catastrophic flood that destroyed the Tongmai Bridge, 17.6 km downstream from the landslide dam. The flood surged downstream through 500 km of Arunachal Pradesh to reach the floodplain of the Brahmaputra River in Assam^12^, India, destroying 20 bridges and leaving 50,000 people homeless. The flood down the gorge produced sustained high bed shear stresses capable of plucking meter-scale blocks from the riverbed^19^.

A prior landslide involving collapse of 5 × 10^8^ m^3^ occurred along Zhamunong gully in 1900^11,18^, and also blocked and dammed the Yigong Tsangpo River, producing a 51.9 km^2^ lake. One month later, the dam collapsed due to lake overtopping, reportedly releasing about 2.7 × 10^9^ m^3^ of water^20^. Investigations and studies on the Yigong landslide concluded that the 2000 landslide eroded portions of the 1900 landslide^17,21^.

## Methods

### Geological age dating

Field surveys examined stratigraphic exposures along Zhamunong gully, as well as in riverbank and valley wall exposures. Stratigraphic exposures in the study area were dated with seven radiocarbon (^14^C) dates on organic materials sampled from the deposits. Field surveys located datable materials at three sites examined in further detail.

### Geomorphic analysis

To examine the influence of repeated landslide dam formation on river channel development, we extracted the longitudinal profile of the Yigong Tsangpo River using a 30 m SRTM (Shuttle Radar Topography Mission) digital elevation model (DEM) and the Google Earth image (Landsat data), based on field investigation aided by the interpretation of 1:25,000 topographic maps. The relative steepness of channel reaches was quantified via the empirical relationship

1 $$ {\text{S}} = {\text{K}}_{{{\text{sn}}}} {\text{A}}^{ - \uptheta } , $$

where *S* is channel slope, *K*_*sn*_ is the steepness index, *A* is upstream area, and *θ* is the concavity index^22^. We used *θ* = 0.45 to calculate *K*_*sn*_ values every km along the river profile.

## Results

Exposures at three sites record emplacement of lacustrine sediments and debris flow deposits several times during the Holocene. Sites 1 and 2 are exposures in the eroded walls of Zhamunong gully (Fig. 1a,c). Site 3 is exposed in valley wall sediments several km up valley from the gully mouth (Fig. 1a).

### Site 1

**Figure 2:**
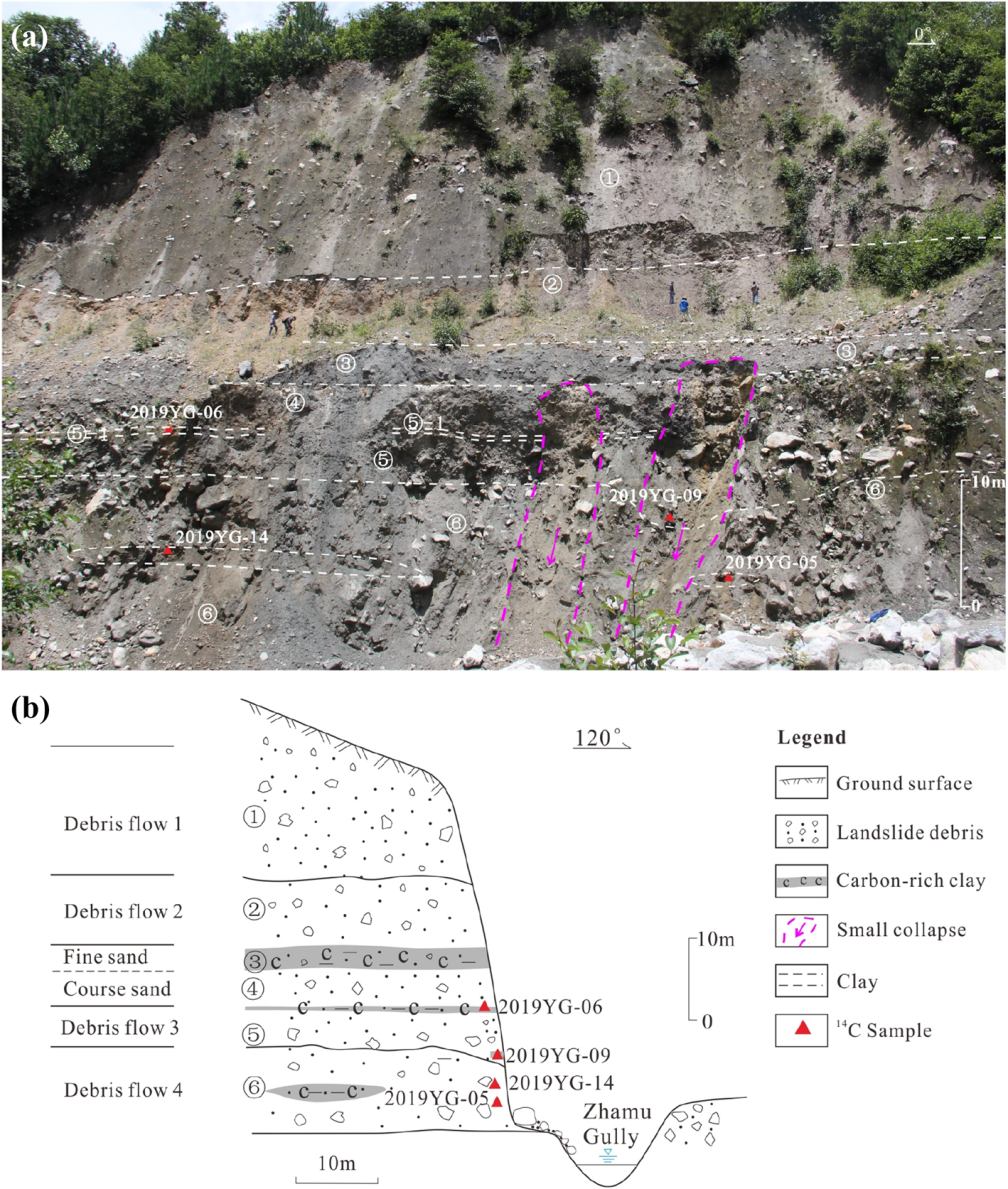
(**a**) Photograph (note people for scale) and (**b**) sketch log of positions of dated samples at Site 1. ① Dark-grey debris flow deposit with gravelly and sandy lenses; ② grayish yellow medium to fine grained debris flow deposit; ③ blue-grey medium to fine grained sand with gravel; ④ grayish-yellow medium coarse sand; ⑤ unsorted debris flow, the top of which consists of carbon-rich peat and charcoal, location of ^14^C samples 2019YG-06 (2520 ± 30 bp) and 2019YG-09 (2450 ± 30 bp); ⑥ debris flow deposit with carbon-rich sandy lenses, location of ^14^C samples 2019YG-14 (3110 ± 30 bp) and 2019YG-05 (3100 ± 30 bp). Figure generated with CorelDRAW X7 software (from https://www.coreldraw.com).

Site 1 is located on the west side of the Zhamunong Gully (Fig. 1c), where a series of four debris flow deposits are exposed in a 50 m-thick section consisting of six identifiable strata (Fig. 2). The uppermost layer consists of a 20.5 m thick massive, unsorted debris flow deposit with gravelly and sandy lenses. This unit unconformably overlies a thinner, older debris flow deposit that displays oxidation extending down from its upper surface, indicating a period of ground surface stability and incipient soil formation prior to emplacement of the overlying debris flow. Below this second debris flow unit several meters of blue-gray medium to fine grained sand overlie up to 5 m of grayish-yellow medium to coarse grained sand. These sands are deposited on top of another (third) unsorted debris flow deposit from which two samples were collected for ^14^C dating, yielding mutually consistent ages of 2450 ± 30 bp (595–411 cal bc) and 2520 ± 30 bp (695–542 cal bc). The lowest unit at the exposure is a fourth debris flow deposit that yielded two consistent ^14^C dates of 3100 ± 30 bp (1432–1283 cal bc) and 3110 ± 30 bp (1437–1288 cal bc). Hence, this exposure reveals evidence for four pre-historic landslides, with one occurring around 1300 bc, another around 600 bc and two since that time (and prior to 1900).

### Site 2

**Figure 3:**
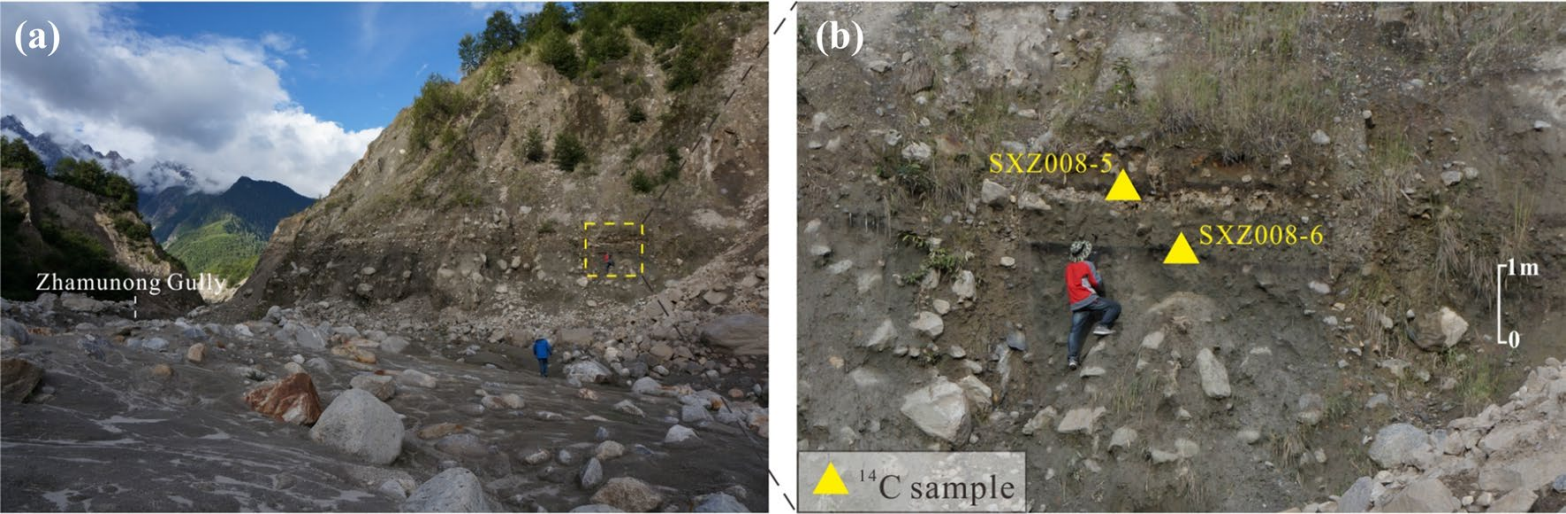
Exposure at Site 2 indicating position of dated samples from the east bank of Zhamunong Gully. (**a**) Context view showing area from which samples were collected. (**b**) Location of ^14^C samples SXZ008-5 (2760 ± 30 bp) and SXZ008-6 (2940 ± 30 bp). Figure generated with CorelDRAW X7 software (from https://www.coreldraw.com).

Two samples of organic debris collected from within the debris flow deposit at Site 2 (Fig. 3) dated at 2760 ± 30 bp (975–830 cal bc) and 2940 ± 30 bp (1225–1045 cal bc). These dates are intermediate in age between the two dated debris flows at Site 1. This deposit likely therefore represents a different landslide event than the two youngest, undated debris flow deposits at Site 1. Hence, there was also at least one pre-historic landslide around 1000 bc.

### Site 3

**Figure 4:**
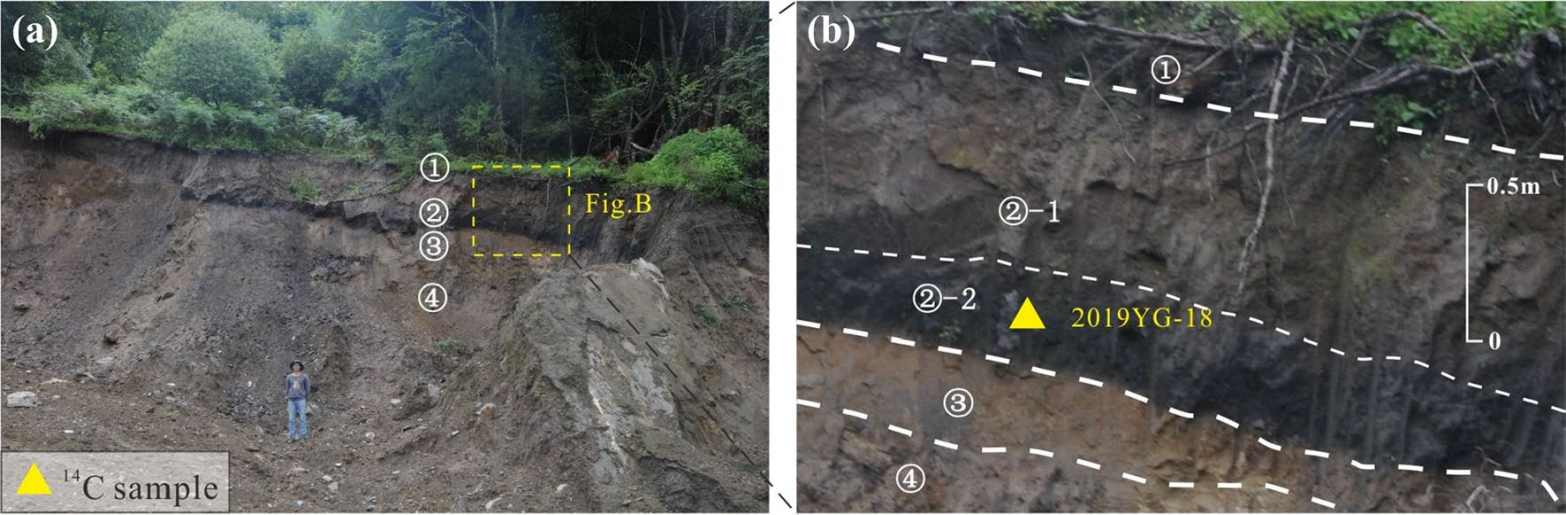
(**a**) Photograph and (**b**) sketch log of exposures at Site 3, showing stratigraphy and position of dated samples: ① Soil, ② lacustrine sediment, ③ weathering surface of ④ debris flow sediment. We collected one wood charcoal sample at depths of 1.2 m (2019YG-18) that yielded a ^14^C calibrated age of 4650 ± 30 years bp (Table 1). Figure generated with CorelDRAW X7 (https://www.coreldraw.com).

Site 3 exposes a section about 8 m thick southeast of Tongjia village, Yigong Town. Field surveys revealed that the section at Site 3 is composed of four distinct layers (Fig. 4a). The surficial layer consists of a roughly 0.5 m thick brown colluvial soil. Below this layer a locally more than 1 m thick deposit of lacustrine sediment consisting of light gray clay rests atop a 30 cm thick layer of organic-matter rich black silty clay (Fig. 4b). A sample of charcoal taken from this layer was ^14^C dated at 4650 ± 30 bp (3517–3396 cal bc). This layer of lacustrine sediment was deposited on top of clay and debris flow sediment, roughly 2 m below the surface.

**Table 1:** ^14^C dating for samples from Yigong landslide-dammed lake sediments.

Field No.	Position	δ^13^C (‰)	Material	Conventional ^14^C age	Calendar calibrated results
2019YG-05	Site 1	− 23.1	Black wood charcoal	3100 ± 30 bp	1432–1283 cal bc (95.4% probability)
2019YG-14	Site 1	− 19.0	Black carbon-rich peat	3110 ± 30 bp	1437–1288 cal bc (95.4% probability)
2019YG-06	Site 1	− 16.8	Black carbon-rich peat	2520 ± 30 bp	694—542 cal bc (65.1% probability)
2019YG-09	Site 1	− 16.6	Black carbon-rich charcoal	2450 ± 30 bp	595–411 cal bc (53.3% probability)
SXZ008-5	Site 2	− 21.8	Black carbon-rich peat	2760 ± 30^a^ bp	975–830 cal bc (68% probability)
SXZ008-6	Site 2	− 21.4	Black carbon-rich peat	2940 ± 30^a^ bp	1225–1045 cal bc (68% probability)
2019YG-18	Site 3	− 21.8	Black wood charcoal (peat)	4650 ± 30 bp	3517–3396 cal bc (80.9% probability)

^a^The ^14^C sample tests were conducted by Beta Analytic, Florida, USA; all samples AMS-Standard delivery pretreated.

### Longitudinal profile

**Figure 5:**
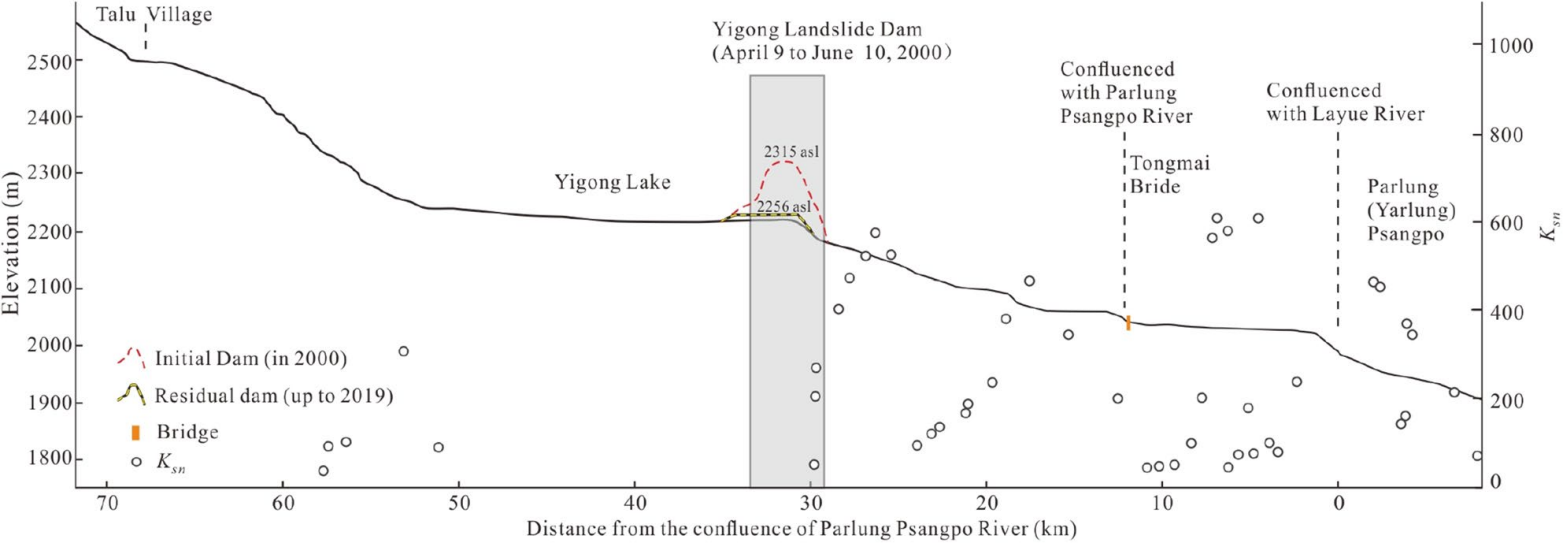
Longitudinal profile for Yigong Psangpo River and the dam created by the 2000 Yigong landslide.

The Yigong landslide dam occurs at the downstream end of a 20 km long reach of the river that has a very low *K*_*sn*_ value due to sediment infilling of the valley bottom (Fig. 5). The more than fourfold drop in *K*_*sn*_ values in the reach upstream of the recurrent dam location indicates little potential for river incision, and thereby headward erosion in this location at present.

## Discussion and conclusions

Historical records, the most reliable sources of data for reconstructing the landslide reoccurrence, are only available for the two most recent (1900 and 2000) landslides. Geological dating of pre-historic landslide deposits generally relies on either ^14^C dating, of organic materials or optically stimulated luminescence (OSL) dating methods of mineral grains^23^. Uncertainties inherent to ^14^C dating include both the analytical error in the reported date and the uncertainty arising from the nature of dated material^24,25^. In radiocarbon dating of charcoal, for example, there is uncertainty associated with the potential for it to have been reworked and whether it came from the inner or outer wood of a tree. While it is preferable to radiocarbon date twigs, cones, or needles the outcrops examined in this study yielded charcoal, black carbon-rich peat and clay for dating the Yigong landslide. Hence, the charcoal samples provide limiting maximum ages for the landslide deposit they were recovered from, as the dated organic material may have died prior to the landslide and been incorporated into it. In contrast, the peaty deposits, like the organic-rich clay, may develop after deposition of the associated sediments, and therefore represent minimum limiting ages. However, the reasonable concurrence of the replicate ages for the samples of each of the landslide deposits reported here suggests that the landslides at sites 1 and 2 represent discrete events reasonably well dated by these ^14^C ages.

**Figure 6:**
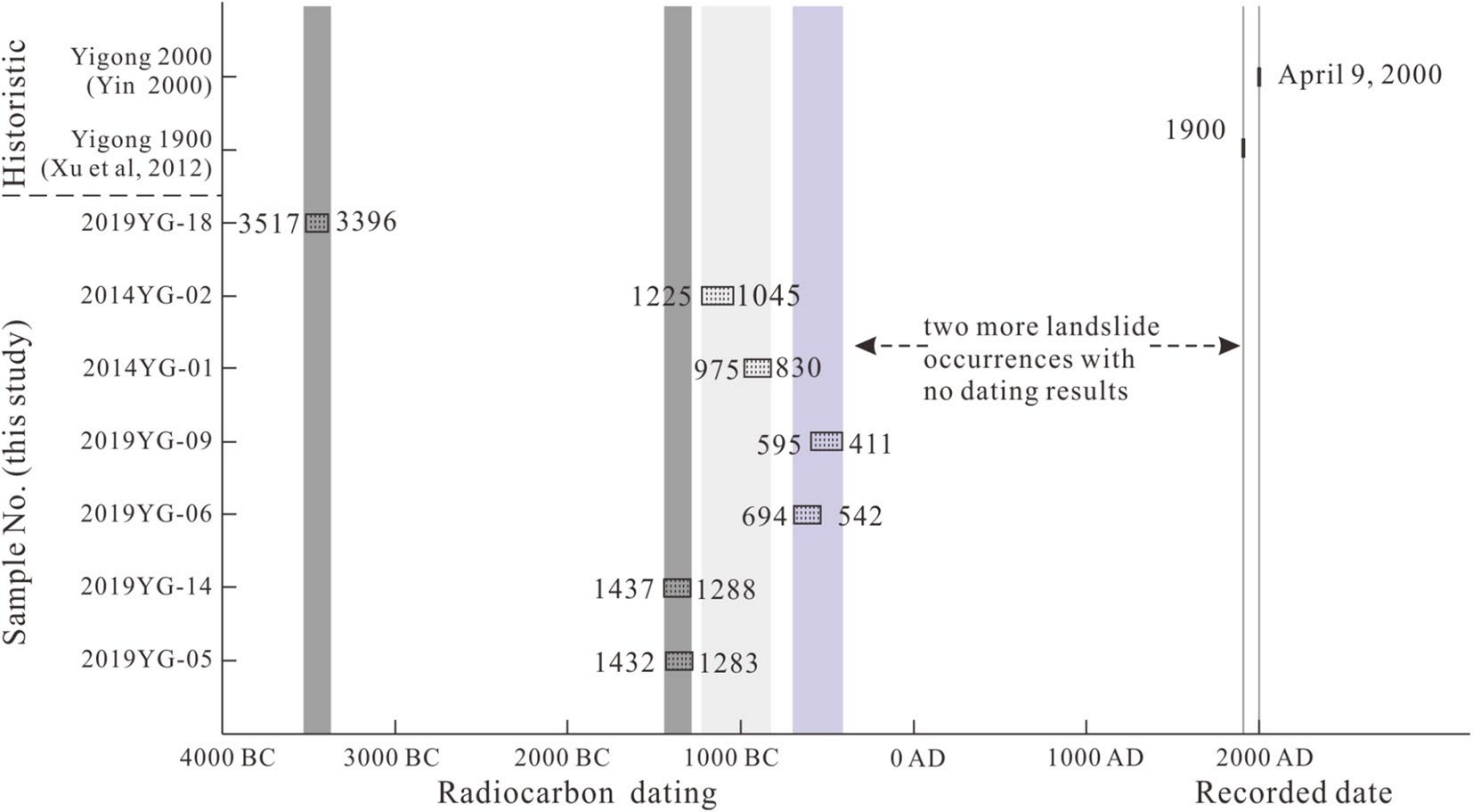
Age ranges dating the different landslides at the Yigong location; width of boxes shown corresponding to ^14^C Calendar calibrated results (this study).

The evidence for repeated landslide-damming of the Yigong Tsangpo River at the location of the 2000 landslide and outburst flood indicates that such impoundments and the floods they produced were recurrent events that occurred frequently enough to potentially influence river profile development. In particular, the new dates for prior late Holocene river-damming landslides allow estimating maximum recurrence intervals for such events. Together with prior reports of the 2000 and 1900 landslides, the exposures of six older landslide deposits indicate that there were at least eight large landslide events at the Yigong landslide over the past 5,500 years (Fig. 6). In light of the dating uncertainties, all together our evidence indicates less than millennial recurrence intervals for river-damming landslides at this location. However, the cluster of dated exposures at sites 1 and 2 indicate that the interval between landslides was just over 200 years across the time interval the two outcrops span, and the two most recent, historical landslides occurred just a century apart (in 1900 and 2000). Hence, the known record of past landslides in this location indicates that channel-damming events occurred with sub-millennia recurrence intervals through the late Holocene.

The uplift of the Tibetan Plateau is the main driving force for topographic development and geomorphic change in this region^26,27^. In the EHSs, river profile steepness values predict that steep fluvial knick points at the southeastern plateau margin should erode rapidly^27^, driving a wave of incision back into the plateau^2^. However, dams that reform or persist at the same location could stabilize knickpoints due to the long-term effects of sedimentation, and recurrent landside dams could stall fluvial kinckpoint retreat, headward incision, and reorganization of the river network^7^. The recurrent damming of the Yigong Tsangpo at the same location over the late Holocene reported here establishes that the Yigong Landslide repeatedly created river-damming deposits capable of retarding upstream incision and potentially producing highly erosive flooding downstream.

At the head of the knickzones on each of the Yarlung Tsangpo, Yigong Tsangpo, and Lulang rivers, landslide or glacial dams acted to retard headward incision over geomorphically significant time scales. For the Lulang landslide, where a large landslide dammed a small tributary, the blockage persisted for millennia through the last glacial maximum as well into the early Holocene^10^. In the case of the Yigong landslide, the direct impoundment by the 2000 landslide only lasted several months, but the sediment impoundment upstream of the landslide location was continuous from 1900 to 2000, and likely persisted between earlier landslides documented here as well. On the Tsangpo River, repeated glacial damming at the same location likewise acted to retard river incision. In each of these locations, the repeated blocking of the river at the same location for a large proportion of time would act to inhibit knickzone propagation and thereby retard river incision into the margin of the Tibetan Plateau^2^.

## References

[1] Cruden DM, Varnes DJ, Turner AK, Shuster RL (1996). Landslide types and processes. Landslides: Investigation and Mitigation.

[2] Korup O, Montgomery DR (2008). Tibetan plateau river incision inhibited by glacial stabilization of the Tsangpo gorge. Nature.

[3] Schmidt KM, Montgomery DR (1995). Limits to relief. Science.

[4] Burbank D, Leland J, Fielding E, Anderson RS, Brozovic N, Reid MR, Duncan C (1996). Bedrock incision, rock uplift and threshold hillslopes in the northwestern Himalayas. Nature.

[5] Montgomery DR, Brandon MT (2002). Non-linear controls on erosion rates in tectonically active mountain ranges. Earth Planet. Sci. Lett..

[6] Costa JE, Schuster RL (1988). The formation and failure of natural dams. Geol. Soc. Am. Bull..

[7] Fan XM, Dufresne A, Subramanian SS, Strom A, Hermanns R, Stefanelli CT, Hewitt K, Yunus AP, Dunning S, Capra L, Geertsema M, Miller B, Casagli N, Jansen JD, Xu Q (2020). The formation and impact of landslide dams—State of the art. Earth Sci. Rev..

[8] Sklar LS, Dietrich WE (2001). Sediment and rock strength controls on river incision into bedrock. Geology.

[9] Montgomery DR, Hallet B, Yuping L, Finnegan N, Anders A, Gillespie A, Greenberg HM (2004). Evidence for Holocene mega floods down the Tsangpo River gorge, southeastern Tibet. Quat. Res..

[10] Wang H, Cui P, Liu DZ, Liu WM, Bazai NA, Wang J, Zhang GT, Lei Y (2019). Evolution of a landslide-dammed lake on the southeastern Tibetan Plateau and its influence on river longitudinal profiles. Geomorphology.

[11] Yin YP (2000). Characteristics of Bomi-Yigong huge high speed landslide in Tibet and the research on disaster prevention. Hydrogeol. Eng. Geol..

[12] Delaney KB, Evans SG (2015). The 2000 Yigong landslide (Tibetan Plateau), rockslide-dammed lake and outburst flood: Review, remote sensing analysis, and process modeling. Geomorphology.

[13] Korup O, Montgomery DR, Hewitt K (2010). Contrasting styles of natural dams at the Tibetan Plateau margin in rivers draining the Himalayan syntaxes. Proc. Natl. Acad. Sci..

[14] Hodges KV (2000). Tectonics of the Himalaya and southern Tibet from two perspectives. Geol. Soc. Am. Bull..

[15] Zhou CH, Yue ZQ, Lee CF, Zhu BQ, Wang ZH (2001). Satellite image analysis of a huge landslide at Yi Gong, Tibet, China. Quart. J. Eng. Geol. Hydrol..

[16] Yin YP, Xing AG (2012). Aerodynamic modeling of the Yigong gigantic rock slide-debris avalanche, Tibet, China. Bull. Eng. Geol. Environ..

[17] Xu Q, Shang YJ, van Asch T, Wang ST, Zhang ZY, Dong XJ (2012). Observations from the large, rapid Yigong rock slide—Debris avalanche, southeast Tibet. Can. Geotech. J..

[18] Shang Y, Yang Z, Li L, Liu DA, Liao Q, Wang Y (2003). A super-large landslide in Tibet in 2000: Background, occurrence, disaster, and origin. Geomorphology.

[19] Turzewski MD, Huntington KW, LeVeque RJ (2019). The geomorphic impact of outburst floods: Integrating observations and numerical simulations of the 2000 Yigong flood, eastern Himalaya. J. Geophys. Res. Earth Surf..

[20] Zhu PY, Wang CH, Wang YC (2003). Large-scale landslide-debris avalanche in Tibet, China Formation of an exceptionally serious outburst flood from a landslide dam in Tibet. Landsl. News..

[21] Zhou J, Cui P, Hao M (2015). Comprehensive analyses of the initiation and entrainment processes of the 2000 Yigong catastrophic landslide in Tibet, China. Landslides..

[22] Korup O (2006). Rock-slope failure and the river long profile. Geology.

[23] Lang A, Moya J, Corominas J, Schrott L, Dikau R (1999). Classic and new dating methods for assessing the temporal occurrence of mass movements. Geomorphology.

[24] Gavin DG (2001). Estimation of inbuilt age in radiocarbon ages of soil charcoal for fire history studies. Radiocarbon.

[25] Jull AJT, Geertsema M (2006). Over 16,000 years of fire frequency determined from AMS radiocarbon dating of soil charcoal in an alluvial fan at bear flat, Northeastern British Columbia. Radiocarbon.

[26] Zeitler PK, Meltzer AS, Koons PO, Craw D, Hallet B, Chamberlain CP, Kidd WSF, Park SK, Seeber L, Bishop M, Shroder J (2001). Erosion, Himalayan geodynamics, and the geomorphology of metamorphism. GSA Today.

[27] Wang YZ, Zhang HP, Zheng DW, Von Dassow W, Zhang ZQ, Yu JJ, Pang JZ (2017). How a stationary knickpoint is sustained: New insights into the formation of the deep Yarlung Tsangpo Gorge. Geomorphology.

